# Oral manifestations of COVID-19 vaccinated individuals, post-infection, and different variants: a Brazilian population study

**DOI:** 10.1590/1807-3107bor-2025.vol39.078

**Published:** 2025-08-04

**Authors:** Larissa Di Carvalho MELO, Juliana Amorim dos SANTOS, Bruna Bastos SILVEIRA, Vitória Tavares de CASTRO, Ana Gabriela Costa NORMANDO, Ana Carolina PRADO-RIBEIRO, Alan Roger SANTOS-SILVA, Fabiana VARGAS-FERREIRA, Eliete Neves Silva GUERRA

**Affiliations:** (a) Universidade de Brasília - UnB, School of Health Sciences, Department of Dentistry, Brasília, DF, Brazil.; (b) Universidade Estadual de Campinas – Unicamp, Piracicaba Dental School, Department of Oral Diagnosis, Piracicaba, SP, Brazil.; (c) Universidade Federal de Minas Gerais – UFMG, School of Dentistry, Department of Social and Preventive Dentistry, Belo Horizonte, MG, Brazil.

**Keywords:** COVID-19, Post-acute COVID-19 syndrome, Surveys and Questionnaires

## Abstract

This cross-sectional study aims to investigate the prevalence of oral manifestations in a sample of the Brazilian population with COVID-19. Adults diagnosed with COVID-19 through real-time PCR/serological tests were invited to participate. The online questionnaires were distributed at different times to analyze and compare SARS-CoV-2 variants considering the period of prevalence of these variants in Brazil. A total of 846 participants were included, of whom 539 were diagnosed before the Omicron variant. In total, 47.28% were vaccinated with at least two doses. The prevalence of oral manifestations was 52.6% (95%CI: 49.23–55.95), and the most common manifestations included taste disorder (38.06%; 95%CI: 34.85–41.38), xerostomia (17.61%; 95%CI: 15.19–20.32), and halitosis (11.58%; 95%CI: 9.59–13.92). The prevalence of persistent symptoms in post-COVID-19 was 12.1% (95%CI: 10.0–14.4) for taste disorder and 5.4% (95%CI: 4.1–7.1) for xerostomia. A significant association was found between females and persistent taste disorder (p = 0.0084) and oral manifestation and depression/anxiety (OR = 1.855, 95%CI: 1.267–2.717, p = 0.002), worse oral hygiene (OR = 1.729, 95%CI: 1.189–2.516, p = 0.004), and medication use (OR = 1.630, 95%CI: 1.123–2.367, p = 0.010) (p < 0.0001). In the Alpha, Beta, Gamma, and Delta variants, compared with the Omicron variant, taste disorder and xerostomia were less present when toothbrushing habits remained unchanged or improved (p < 0.0001). Oral manifestations in patients with COVID-19 were associated with depression/anxiety, worse oral hygiene, and medication, all of which reinforce its multifactorial etiopathogenesis.

## Introduction

The respiratory disease caused by the severe acute respiratory syndrome coronavirus 2 (SARS-CoV-2), officially named Coronavirus Disease 2019 (COVID-19), first emerged in late 2019 in Wuhan, China. On March 11, 2020, COVID-19 was declared a pandemic by the World Health Organization (WHO). To date, the estimated number of confirmed cases has reached 775,335,916 worldwide, with 7,045,569 reported deaths (April 29, 2024, WHO data).^1^

The lungs serve as the primary sites of SARS-CoV-2 infection, and the most common symptoms are fever, dry cough, and dyspnea. Also, there have been reports of oral manifestations, including taste disorders and xerostomia.^2-5^ The oral cavity (mainly the nose and the eyes) constitutes a significant portal of entry for SARS-CoV-2.^6^ This can be attributed to the expression of angiotensin 2 converting enzyme (ACE2), TMPRSS2, and furin in salivary glands and oral mucosal epithelial cells, which act as receptors for viral cell entry.^3,6^ Studies have also detected the presence of the virus in the saliva of infected individuals.^7^ However, there remain gaps in our understanding of the immune response, and the precise role of the oral cavity in characterizing the disease is yet to be fully elucidated.

Regarding the oral manifestations associated with COVID-19, it remains uncertain whether they are a direct result of viral infection by SARS-CoV-2 or whether they are secondary manifestations and coinfections resulting from systemic consequences.^8^ In addition to viral invasion, other factors, such as suboptimal oral hygiene, stress, or dysbiosis due to therapeutic drugs, are speculated to contribute to these symptoms.^9^ Observations have included halitosis, ulcerations, sialadenitis, and a dry mouth sensation, possibly linked to reduced salivary flow.^9-12^ Taste disorders and xerostomia were reported to be concurrent with COVID-19 infection and are among the earliest symptoms in the progression of this disease.^13^ Thus, these disorders may be more prevalent in mild and moderate cases and can be readily identified, offering potential for intervention, particularly in areas with limited access to widespread testing.^11^

Numerous studies have investigated oral manifestations in COVID-19 patients. Nevertheless, there are still crucial unanswered questions: a) Does COVID-19 vaccination prevent the occurrence of oral lesions? b) Do symptoms such as xerostomia or taste changes persist for at least 15 days post-COVID-19 infection? c) Are there differences in oral manifestations between SARS-CoV-2 variants? d) Does the Omicron variant of SARS-CoV-2 exhibit variations in the occurrence and types of oral manifestations compared to earlier variants? Therefore, it is essential to comprehend and monitor the occurrence of oral manifestations concerning vaccination, viral variants, and the persistence of COVID-19-related symptoms. In this context, assessment of the oral cavity is extremely important, allowing the dental team to contribute substantially not only to the care of affected patients but also to the identification of the initial and/or prevalent manifestations. Additionally, this assessment provides a deeper understanding of the etiopathogenesis of these manifestations and fosters scientific advancements in the understanding of COVID-19 in the post-pandemic period.^9^

It is crucial to investigate how both the progression of the pandemic and vaccination status may influence the prevalence and persistence of oral symptoms. In this context, self-reported data from individuals across different periods of the pandemic can provide valuable insights into these manifestations. Therefore, this cross-sectional study aimed to investigate, through an online questionnaire, the prevalence and persistence of oral manifestations in individuals with COVID-19 during two distinct periods of the pandemic: before and after the emergence of the Omicron variant, with the primary focus on understanding the overall impact of COVID-19 and its variants on oral health. The secondary objective of this study was to compare the outcomes between vaccinated and non-vaccinated populations.

## Methods

### Study design and ethical considerations

This cross-sectional study was an online questionnaire-based survey conducted in two steps to evaluate the prevalence of oral manifestations in a Brazilian sample affected by COVID-19. The survey was distributed at two different time points: from October 2021 to December 2021 (before the Omicron variant emerged), and from June 2022 to July 2022 (during the Omicron outbreak in Brazil). The questionnaires were distributed at different times to analyze and compare the variants of COVID-19 (Alpha, Beta, Gamma, and Delta variants versus the Omicron variant), considering the initial detection dates of these variants.^14^ The criterion for participation in the second phase was having contracted COVID-19 exclusively in 2022. This project was approved by the local ethics committee of the Faculty of Health Sciences at the University of Brasília in accordance with the Declaration of Helsinki for Human Research (CAAE: 48224221.2.0000.0030). All participants received information about the study and provided online consent. Participation was voluntary, and participants could withdraw at any time while ensuring the confidentiality of their identity.

### Sample and data collection

The target sample consisted of participants aged over 18 years who had been diagnosed with COVID-19 through either real-time PCR or serological tests and who consented to participate in the study. The questionnaire was accessible on various internet-connected devices, including cell phones, computers, and tablets. The questionnaire was distributed via email and promoted on social media platforms such as Instagram®, Facebook®, and WhatsApp®. The sample size was determined by estimating the number of the Brazilian population with a margin of error of 5% and a 95% confidence interval, using the OpenEpi online calculator. The parameters used to calculate the sample size were 50% prevalence of the outcome, 95% confidence interval, and 80% power. The study sample should include at least 384 participants. An additional 20% was included to account for potential losses and refusals, yielding a total of 460 participants.

### Questionnaire

The online questionnaire was generated in the Google Forms application (Google Search, Melon Park, United States) and was based on questions formulated by the research team. In addition to these questions, externally validated questions on xerostomia were incorporated from the Summated Xerostomia Inventory-Dutch Version.^15^ For internal validity, the questionnaire was pre-assessed by a research group with expertise in oral manifestations of COVID-19^16^ before it was distributed. The questionnaire was made available in Brazilian Portuguese, and participants were required to consent before proceeding; otherwise, data collection was automatically stopped. The questions were split into six sections: (a) Demographic information of the participants, (b) Signs and symptoms of COVID-19, (d) Patient’s oral hygiene status, (d) Oral manifestations presented during COVID-19 infection, (e) Xerostomia, and (f) Persistence of symptoms within a period of 15 days after the infection had resolved (Table 1). A period of 15 days after infection recovery was considered because reports on “post-COVID-19 syndrome” vary from 15 days to 12 weeks with no standardized consensus.^17^ All responses were self-reported by the participants through the questionnaire. This includes self-reported classifications of smoking status (smokers vs. non-smokers), alcohol consumption (alcohol consumer vs. non-alcohol consumer), and oral hygiene status. Regarding oral hygiene, participants were asked to indicate whether the quality of their toothbrushing worsened, improved, or remained unchanged during the COVID-19 infection.

**Table 1:** Full set of items included in the applied questionnaire survey.

Variables
General characterization
Which test was used for your diagnosis of COVID-19? You can select more than one option
Real-time PCR (nasopharyngeal swab collection)
Serological test/blood test/rapid test
Chest tomography
Nasal swab immunochromatography
I did not do a COVID-19 diagnostic test
Others: To specify
Female
Male
Not declared
AC
AL
AP
AM
BA
CE
DF
ES
GO
MA
MT
MS
MG
PA
PB
PR
PE
PI
RJ
RN
RS
RO
RR
SC
SP
SE
TO
Less than 1 minimum wage
1 minimum wage
2 to 4 minimum wages
5 or more minimum wages
What is your occupation?
Healthcare professional
Students in the health field
I am not a health professional or a health student
Yellow
White
Brown
Black
Indigenous
Not declared
I have diabetes and/or take medication for diabetes
I have high blood pressure (hypertension) and/or take medicine for high blood pressure
I have high cholesterol or triglycerides (dyslipidemia) and/or take cholesterol medication
I have cirrhosis of the liver and/or liver fat and/or hepatitis
I have kidney problems (my kidney doesn’t work very well)
I have heart problems: arrhythmia, heart attack, angina, valve, pacemaker
I have lung problems: asthma, bronchitis, chronic obstructive pulmonary disease (COPD)
I have thyroid problems and/or take thyroid medication
I have depression and/or anxiety and/or take medication for depression/anxiety
I have epilepsy and/or take medication for epilepsy
I have lupus
I have rheumatoid arthritis
I have Sjögren’s syndrome
I have HIV/AIDS
I am transplanted (liver, kidney, lung, and/or heart)
I have graft versus host disease (GVHD)
I have had cancer and needed chemotherapy
I had or am undergoing radiotherapy in the head and neck region
I have cystic fibrosis
I have Bell’s palsy
I have amyloidosis
I have sarcoidosis/Besnier-Boeck disease
I have hepatitis C
I have anorexia and/or I take an antianorexic drug
I don’t have any disease
Other: To specify
Yes
No
Former smoker
Never
Occasionally
Often
Yes
No
Former alcoholic
Never
Occasionally
Often
Fever
Dry cough
Coryza (runny nose)
Sore throat
Felt unwell
Headache
Nausea, vomiting, or diarrhea
Change in smell (smell)
Change in taste (taste/flavor)
Lack of appetite (desire to eat)
Muscle pain
Difficulty breathing or shortness of breath
Others: To specify
I just needed to stay at home
I had to go to the hospital, but I was not hospitalized
I had to go to the hospital and I was hospitalized
I needed to go to the hospital and was admitted to the ICU, but I did not need respirators (such as an oxygen catheter, oxygen mask with passive ventilation, IOT, respiratory physical therapy, etc.)
I needed to go to the hospital, and I also needed the help of respirators/ I was intubated (such as an oxygen catheter, oxygen mask with passive ventilation, IOT, respiratory physical therapy, etc.)
Monoclonal antibodies (teluximab, banlanivimab, etesevimab, eegdanvimab, sotrovimab, etc.)
Corticosteroids (dexamethasone, prednisolone, prednisone, etc.)
Non-steroidal anti-inflammatory drugs (ibuprofen, diclofenac, nimesulide, etc.)
Analgesics (metamizole, acetaminophen, etc.)
Antibiotics (amoxicillin, ampicillin, clindamycin, penicillin, etc.)
Antivirals (rendesevir, etc.)
Chloroquine/Hydroxychloroquine
I did not use any medication
Others: To specify
Have you been vaccinated against COVID-19?
Yes
Not
How many doses of COVID-19 vaccine did you take?
One
Two
Three
Not applicable, as I have not been vaccinated yet
Not applicable, as I refused to take the vaccine
What vaccine against COVID-19 did you take?
AstraZeneca
CoronaVac
Covaxin
Janssen
Modern
Pfizer
Sputnik V
Not applicable, as I have not been vaccinated yet
Not applicable, as I refused to take the vaccine.
Other: To specify
How many days after the second dose did you take the third dose? Please reply in days (Example: 90). If you have not yet taken the third dose or do not fit into the group that needs to take the third dose, please answer with the number zero, “0”
Before being vaccinated
After being vaccinated
Not applicable, as I have not been vaccinated yet
Not applicable, as I refused to take the vaccine
How many times a day do you brush your teeth? Please answer in whole numbers (Example: 2)
Do you use mouthwashes?
Never
Occasionally
Often
Do you notice bleeding when brushing your teeth or gingival bleeding?
Never
Occasionally
Often
Do you do cleaning/scaling with the dentist?
Never
Occasionally
Often
Do you use dental floss/tape?
Never
Occasionally
Often
Do you have mobile teeth?
Yes
No
I do not know
How was your oral hygiene DURING the period of Covid-19 infection?
Better than normal
I kept my oral hygiene in the same way I did before Covid-19
Worse than normal.
Oral manifestations
Did you present any manifestation in the oral cavity DURING the period of infection by COVID-19?
Yes
No
Did you experience any of the following signs or symptoms during COVID-19 infection? You can select more than one option
Bad breath (halitosis)
Oral wounds (ulcerations)
Herpes-like lesions
Fungal infection
Wound(s) in the corner(s) of the mouth (angular cheilitis)
Pain in teeth or mouth
Pain in the maxilla, mandible, or temporomandibular joint (TMJ) region
Pain or swelling in front of the ear (in the parotid gland region)
Pain or swelling below the mandible (in the region of the submandibular glands)
A burning sensation in the mouth or tongue
Tongue redness
Bleeding gums
Change in food taste (taste)
Difficulty in swallowing food
Dry mouth (xerostomia)
I didn’t have any oral signs/symptoms
Others: To specify
If you had any change in the taste of food (taste), how was it?
Partial
Total
Metallic/bitter taste
I had no change in taste
Xerostomia
Regarding the sensation of dry mouth, please select the option that best describes your symptoms BEFORE contracting COVID-19
My mouth feels dry when eating a meal
Never
Occasionally
Often
My mouth feels dry
Never
Occasionally
Often
I have difficulty eating dry foods
Never
Occasionally
Often
I have difficulties swallowing certain foods
Never
Occasionally
Often
My lips feel dry
Never
Occasionally
Often
Regarding the sensation of dry mouth, please select the option that best describes your symptoms DURING contracting COVID-19
My mouth feels dry when eating a meal
Never
Occasionally
Often
My mouth feels dry
Never
Occasionally
Often
I have difficulty eating dry foods
Never
Occasionally
Often
I have difficulties swallowing certain foods
Never
Occasionally
Often
My lips feel dry
Never
Occasionally
Often
Post-COVID-19 (15 days after infection)
I still have difficulty perceiving smells (smell)
Yes
No
Not applicable, as I still have the disease, or I have been cured of the disease recently (less than 15 days)
I still have difficulty perceiving the taste of food (taste)
Yes
No
Not applicable, as I still have the disease, or I have been cured of the disease recently (less than 15 days)
I still have a sensation of dry mouth (xerostomia)
Yes
No
Not applicable, as I still have the disease, or I have been cured of the disease recently (less than 15 days)

### Statistical analyses

Data analysis was performed descriptively, involving the construction of prevalence tables and graphs, with means and 95% confidence intervals (95% CI). Descriptive statistics were calculated to summarize the characteristics of the study population. A binomial logistic regression analysis was conducted to calculate odds ratios (ORs) and 95%CI for the predictor variables to evaluate associations with oral manifestations, focusing on the presence and absence of these manifestations, as well as on the three most prevalent conditions: taste disorder, xerostomia, and halitosis. For the initial model assessing oral manifestations, the following variables were included: age, sex, chronic disease and/or use of controlled medication, depression/anxiety, hypertension, various signs and symptoms (fever, malaise, headache), severity/evolution (respirator use), use of medication during COVID-19 (analgesics, antibiotics, steroidal anti-inflammatory drugs), duration of signs/symptoms, brushing quality, and the Omicron variant. In the initial model for taste disorder, the variables included were age, sex, depression/anxiety, signs and symptoms (malaise, headache), severity/evolution, use of medication during COVID-19 (analgesics, antibiotics), duration of signs/symptoms, brushing quality, and the Omicron variant. For xerostomia, the initial variables considered were sex, chronic disease and/or use of controlled medication, depression/anxiety, smoking, smoking frequency, signs and symptoms (fever, malaise, headache), severity/evolution, respirator use, use of medication during COVID-19 (analgesics, steroidal anti-inflammatory drugs), and brushing quality. Lastly, the model for halitosis included age, sex, chronic disease and/or use of controlled medication, depression/anxiety, hypertension, smoking, smoking frequency, signs and symptoms (fever, malaise, headache), severity/evolution, respirator use, use of medication during COVID-19 (analgesics, antibiotics, steroidal anti-inflammatory drugs), vaccine doses, brushing quality, and the Omicron variant. The final models included only the statistically significant variables for each condition. Normality was assessed using the Shapiro-Wilk and Kolmogorov-Smirnov tests. For comparisons between groups, the chi-square test was employed to evaluate associations between categorical variables. All statistical analyses were performed using GraphPad Prism version 9.0.2 (GraphPad Software, La Jolla, USA) and Jamovi (Version 2.3). The significance level was set at p < 0.05.

## Results

### Participants and demographic data

A total of 1,016 participants consented to participate in the study. Out of these, 170 participants were excluded either because of the lack of COVID-19 diagnostic tests or their age being under 18 years. Thus, 846 participants were included, of whom 539 were assessed before the Omicron variant and 307 after its outbreak. The sample consisted of 620 (73.29%) females, 225 (26.6%) males, and one participant who did not declare their sex (0.12%). The mean (± SD) age was 39.01±13.82 years (range: 18–85 years). The median education level was 18 years (ranging from 3 to 55 years of study), with a mean of 17.76 years (± 6.32). Regarding family income, 71.16% of participants reported earning five or more Brazilian minimum wages (BMW), 23.76% earned between two to four minimum wages, 3.43% earned exactly one minimum wage, and 1.65% earned less than one minimum wage. The median income was 5 BMW, and the mean was 2.6 BMW (± 0.63). In terms of race/ethnicity, 66.9% of participants identified themselves as white, 24.11% as brown, 4.96% as black, 2.84% as yellow, and 1.18% did not declare their race/ethnicity. No participants identified themselves as indigenous. In the regional distribution, 57.57% were from the Midwest, 23.76% from the Southeast, 10.64% from the Northeast, 4.73% from the South and, 3.3% from the North. Almost 47.28% were vaccinated with at least two doses. Moreover, 50.95% were infected by COVID-19 prior to vaccination. As for smoking and alcohol consumption habits, 10.64% were smokers, 83.33% were non-smokers, and 6.03% were former smokers. In terms of alcohol consumption, 74.59% reported consuming alcohol, 25.06% were non-drinkers, and 0.35% were former alcohol consumers. These classifications were based on the participants’ responses to questions about their use of tobacco products and alcohol. Participants who responded negatively to the consumption question were instructed to select “never” for the frequency question. The complete demographic and general data are shown in Table 2.

**Table 2:** General characterization. Distribution of the sample according to socioeconomic and demographic characteristics and oral behaviors in patients with COVID-19 (n = 846).

Variables	n	% (95% CI)
Sex		
Male	225	26.60 (23.73–29.67)
Female	620	73.29 (70.21–76.16)
Not declared	1	0.12 (0.02–0.66)
Age		
	Median: 38 (18–85)	
	Mean: 39.01 (± 13.82)	
Race/ethnicity		
Black	42	4.96 (3.69–6.64)
Brown	204	24.11 (21.35–27.11)
White	566	66.9 (63.60–70.00)
Yellow	24	2.84 (1.90–4.20)
Indigenous	0	0
Not declared	10	1.18 (0.60–2.10)
Region		
North	28	3.30 (2.30–4.74)
Northeast	90	10.64 (8.70–13.00)
Midwest	487	57.57 (54.21–60.85)
Southwest	201	23.76 (21.00–26.70)
South	40	4.73 (3.50–6.40)
Educational level (years of study)		
	Median: 18 (3–55)	
	Mean: 17.76 (±6.32)	
Family Income (BMW*)		
	Median: 5	
	Mean: 2.6 (±0.63)	
Less than 1 minimum wage	14	1.65 (0.98–2.75)
1 minimum wage	29	3.43 (2.39–4.88)
2 to 4 minimum wages	201	23.76 (21.01–26.74)
5 or more minimum wages	602	71.16 (68.02–74.11)
Smoking experience		
Non-smokers	705	83.33 (80.67–85.69)
Smokers	90	10.64 (8.73–12.90)
Former smokers	51	6.03 (4.61–7.84)
Alcohol experience		
Non-alcohol consumer	212	25.06 (22.26–28.09)
Alcohol consumer	631	74.59 (71.55–77.40)
Former alcoholic	3	0.35 (0.12–1.03)
Occupation		
Healthcare student	94	11.11 (9.16–13.41)
Healthcare professional	278	32.86 (29.78–36.10)
Neither healthcare professionals nor student	474	56.03 (52.66–59.34)
Vaccinated		
No	18	2.13 (1.35–3.34)
Yes	828	97.87 (96.66–98.65)
Number of doses of vaccine against COVID-19		
None	18	2.13 (1.35–3.34)
1 dose	59	6.97 (5.44–8.89)
2 doses	400	47.28 (43.94–50.65)
Booster dose	369	43.62 (40.31–46.98)
Moment of COVID-19 infection		
Before being vaccinated	431	50.95 (47.58–54.30)
After being vaccinated	397	46.92 (43.59–50.30)
Not vaccinated	18	2.13 (1.35–3.34)
Severity/evolution of COVID-19		
Mild	810	95.74 (94.17–96.91)
Moderate/severe	36	4.26 (3.09–5.88)
Chronic disease and/or use of controlled medication		
Depression/Anxiety	148	17.49 (15.08–20.20)
Hypertension	99	11.70 (9.70–14.04)
Cholesterol	83	9.81 (7.98–12.00)
Thyroid disorder	65	7.68 (6.07–9.67)
Lung disease	53	6.26 (4.82–8.10)
Diabetes	42	4.96 (3.69–6.64)
Heart disease	21	2.48 (1.62–3.76)
Cirrhosis	20	2.36 (1.53–3.62)
Rheumatoid arthritis	8	0.95 (0.47–1.85)
Other	58	6.86 (5.34–8.76)
No chronic disease	461	54.49 (51.12–57.88)
Signs and symptoms during COVID-19		
Fever	545	64.42 (61.14–67.57)
Malaise	535	63.24 (59.94–66.42)
Headache	494	58.39 (55.04–61.67)
Dry cough	492	58.16 (54.8–61.40)
Coryza	455	53.78 (50.41–57.12)
Smell disorder	429	50.71 (47.34–54.07)
Sore throat	427	50.47 (47.11–53.83)
Muscle pain	400	47.28 (43.94–50.65)
Taste disorder	322	38.06 (34.85–41.38)
Lack of appetite	261	30.85 (27.83–34.04)
Breathlessness	201	23.76 (21.01–26.74)
Nausea. Vomiting, and diarrhea	198	23.40 (20.68–26.37)
Xerostomia	149	17.61 (15.19–20.32)
Halitosis	98	11.58 (9.59–13.92)
Tiredness	25	2.96 (2.01–4.32)
Brain fog	9	1.06 (0.56–2.01)
Other	67	7.92 (6.28–9.93)
No signs and symptoms during COVID-19	25	2.96 (2.01–4.32)
Medication use during COVID-19		
Analgesic	557	65.84 (62.58–68.96)
Antibiotics	295	34.87 (31.73–38.14)
Steroidal anti-inflammatory drugs	280	33.10 (30.01–36.34)
Non-steroidal anti-inflammatory drugs	214	25.30 (22.50–28.30)
Chloroquine / Hydroxychloroquine	75	8.87 (7.13–10.97)
Antiparasitic (Ivermectin)	41	4.85 (3.59–6.51)
Vitamins	13	1.54 (0.90–2.60)
Monoclonal antibodies	9	1.06 (0.50–2.00)
Expectorant	9	1.06 (0.50–2.00)
Antihistamine	7	0.83 (0.40–1.70)
Antivirals	5	0.59 (0.20–1.40)
Opioid	1	0.12 (0.02–0.60)
Other	56	6.62 (5.10–8.50)
Do not know	5	0.59 (0.20–1.40)
No medication use	134	15.84 (13.50–18.40)
Oral hygiene status (before COVID-19)		
Daily brushing frequency	Median: 3	
	Mean: 2.82 (±0.86)	
Mouthwash use		
Occasionally	421	49.76 (46.04–53.13)
Often	77	9.10 (7.34–11.23)
Never	348	41.13 (37.87–44.54)
Gingival bleeding		
Occasionally	348	41.13 (37.87–44.48)
Often	24	2.84 (1.91–4.18)
Never	474	56.03 (52.66–59.34)
Dental prophylaxis		
Occasionally	545	64.42 (61.14–67.57)
Often	191	22.58 (19.89–25.51)
Never	110	13.00 (10.90–15.44)
Flossing		
Occasionally	278	32.86 (29.78–36.10)
Often	515	60.87 (57.54–64.11)
Never	53	6.26 (4.82–8.10)
Tooth mobility		
Yes	51	6.03 (4.61–7.84)
Not reported	192	22.70 (20.00–25.64)
No	603	71.28 (68.14–74.22)
Quality of oral hygiene during COVID-19		
Better or no modifications	764	90.31 (88.13–92.12)
Worse	82	9.69 (7.87–11.87)
Oral manifestations		
Present	445	52.6 (49.23–55.95)
Absent	401	47.4 (44.05–50.77)
Oral manifestations		
Taste disorder	322	38.06 (34.85–41.38)
Xerostomia	149	17.61 (15.19–20.32)
Halitosis	98	11.58 (9.59–13.92)
Difficulty swallowing food	71	8.39 (6.70–10.45)
Pain in the jaw or temporomandibular joint (TMJ) region	69	8.16 (6.49–10.19)
Oral mucosal lesions	55	6.5 (5.02–8.36)
Pain in teeth or mouth	50	5.91 (4.51–7.70)
Salivary gland disorder	37	4.37 (3.19–5.97)
Burning sensation in the mouth or on the tongue	30	3.55 (3.19–5.97)
Bleeding gum	24	3.9 (2.49–5.01)
Angular cheilitis	17	2.01 (1.19–4.18)
Redness on the tongue	11	1.30 (0.72–2.31)
Other	8	0.95 (0.47–1.93)
No oral manifestations	401	47.4 (44.05–50.77)

*Brazilian minimum wage (BMW).

### COVID-19 signs and symptoms

The main signs and symptoms reported during COVID-19 infection were fever (64.42%; 95%CI: 61.14–67.57), malaise (63.24%; 95%CI: 59.94–66.42), headache (58.39%; 95%CI: 55.04–61.67), dry cough (58.16%; 95%CI: 54.8–61.40), coryza (53.78%; 95%CI: 50.41–57.12), smell disorder (50.71%; 95%CI: 47.34–54.07), sore throat (50.47%; 95%CI: 47.11–53.83), muscle pain (47.28%; 95%CI: 43.94–50.65), and taste disorder (38.02%; 95%CI: 34.85–41.38) (Table 2).

### Prevalence of oral manifestations

**Figure 1:**
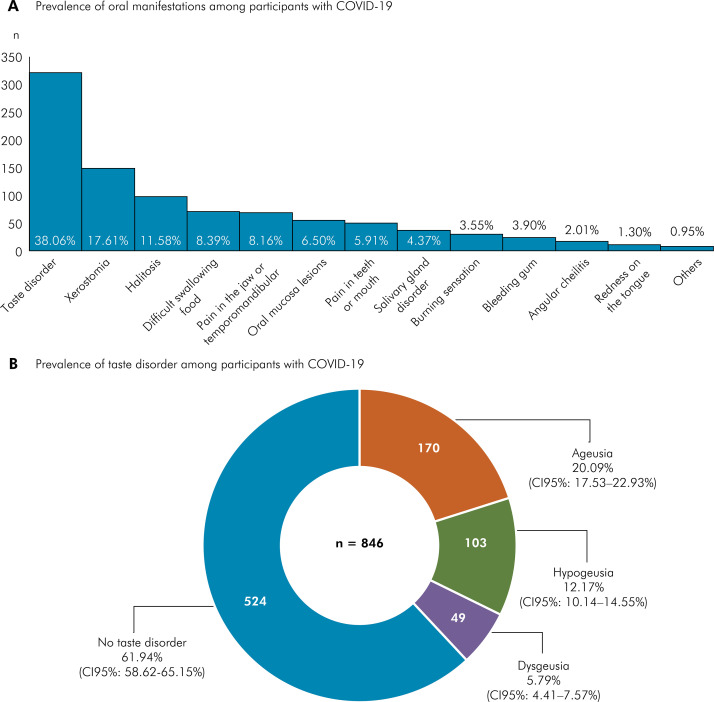
(a) Prevalence of oral manifestations among participants diagnosed with COVID-19; (b) Prevalence of taste disorder among participants diagnosed with COVID-19.

The prevalence of oral manifestations was 52.6% (95%CI: 49.23–55.95), of which taste disorder (38.06%; 95% CI: 34.85–41.38), xerostomia (17.61%; 95%CI: 15.19–20.32), and halitosis (11.58%; 95%CI: 9.59–13.92) were the most frequent ones (Figure 1a). As for those who reported taste disorder, ageusia was observed in 20.09% (95%CI: 17.53–22.93), hypogeusia in 12.17% (95%CI: 10.14–14.55), and dysgeusia in 5.79% (95%CI: 4.41–7.57) (Figure 1b) (Table 2).

### Oral manifestations, chronic diseases, and medication use during COVID-19 infection

**Figure 2:**
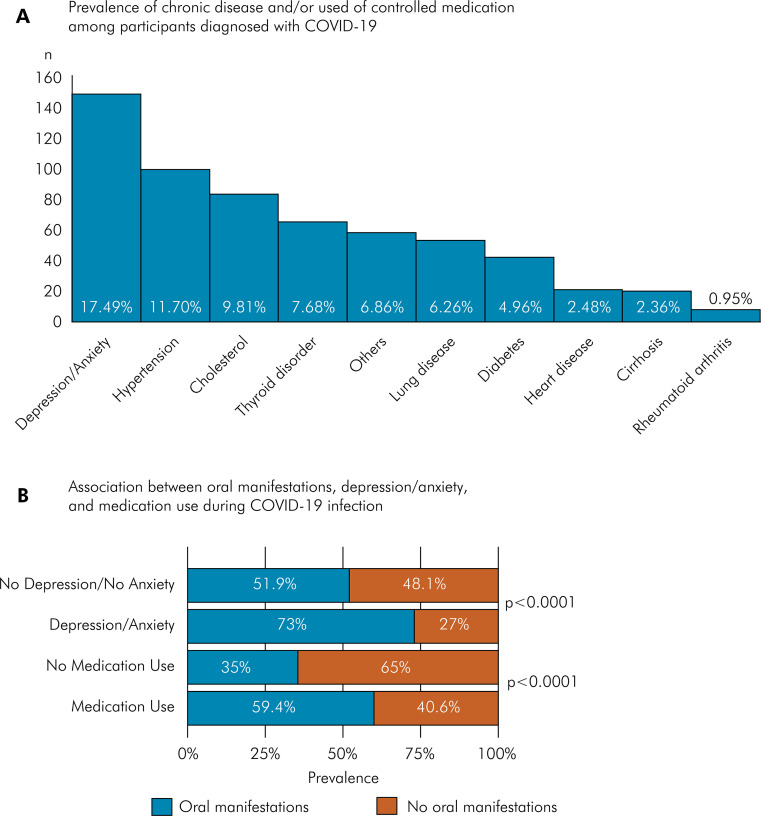
(a) Prevalence of chronic disease and/or used controlled medication among participants diagnosed with COVID-19; (b) Association between oral manifestations, depression/anxiety (p < 0.0001), and medication use during COVID-19 infection (p < 0.0001).

The most common chronic condition reported was depression and anxiety (17.49%; 95%CI: 15.08–20.20) (Figure 2a). An association was found between depression/anxiety and oral manifestations (p < 0.0001). Regarding medication use, 84.16% were on medication, of which analgesics (65.84%; 95%CI: 62.58–68.96), antibiotics (34.87%; 95%CI: 31.73–38.14), and steroidal anti-inflammatory drugs (33.1%; 95% CI: 30.01–36.34) were the most frequently reported. The use of chloroquine/hydroxychloroquine (8.87%; 95% CI: 7.13–10.97) and antiparasitic drugs, such as ivermectin (4.85%; 95%CI: 3.59–6.51) was also reported. In addition, there was a higher prevalence of oral manifestations among those who were taking any medication during COVID-19 infection (p < 0.0001) (Figure 2b) (Table 2).

### Oral manifestations, sex, and quality of oral hygiene during COVID-19 infection

**Figure 3:**
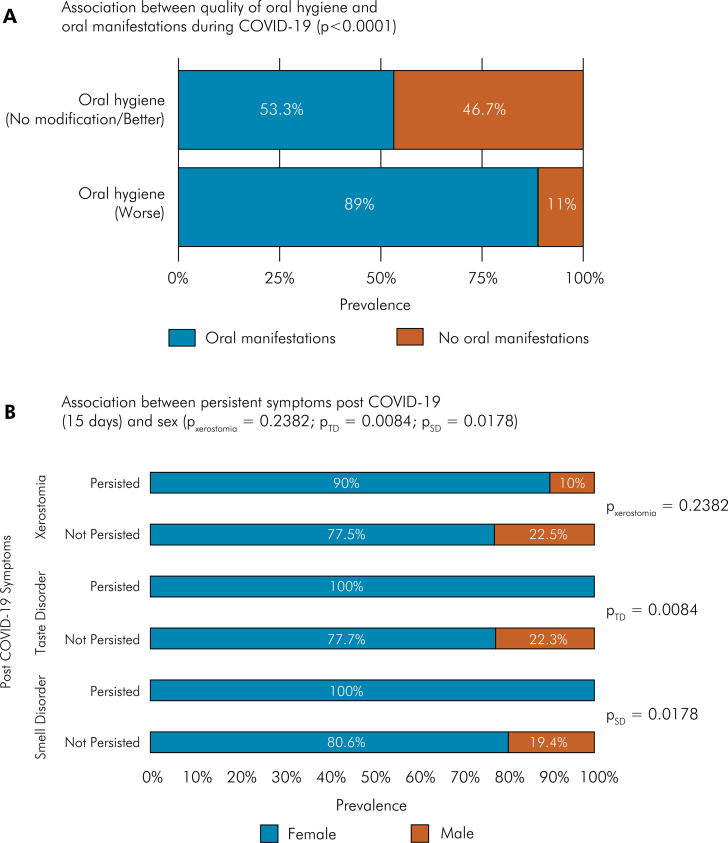
(a) Prevalence of oral manifestations in comparison with the quality of oral hygiene during COVID-19 infection with statistical significance between oral manifestation and worse oral hygiene status (p < 0.0001); (b) Prevalence of persistent symptoms post-COVID-19 (15 days) and sex. Significant association between female participants and persistent smell disorder (pSD = 0.0178) and persistent taste disorder (pTD = 0.0084) for post-COVID-19 symptom persistence . No significant association between sex and persistent xerostomia (pxerostomia = 0.2382).

A significant association was observed between females and oral manifestations (p < 0.0001). During the COVID-19 pandemic, 90.31% of participants reported that their oral hygiene was better or showed no modifications, while 9.69% indicated a decline in their oral hygiene quality. Oral manifestations were more prevalent among those who had a worse oral hygiene (p < 0.0001), especially related to halitosis (p = 0.0395) and worse brushing quality (Figure 3a). Regarding oral hygiene status before COVID-19, the median daily brushing frequency was three times a day, with a mean of 2.82 (± 0.86). In terms of mouthwash use, 49.76% of participants reported using it occasionally, 9.10% used it often, and 41.13% never used it. Gingival bleeding occurred occasionally in 41.13% of participants, often in 2.84%, and never in 56.03%. Dental prophylaxis was performed occasionally by 64.42% of participants, often by 22.58%, and never by 13%. Flossing was done occasionally by 32.86%, often by 60.87%, and never by 6.26%. Tooth mobility was reported by 6.03% of participants, while 71.28% reported no mobility, and 22.70% had no related issues. No significant statistical differences were observed between the quality of oral hygiene and xerostomia (p = 0.637), taste disorder (p = 0.435), or oral mucosal lesions (p = 0.115) (Table 2).

### Oral manifestations, vaccination, and severity of COVID-19 infection

No statistically significant association was detected between oral manifestations and vaccination status (p = 0.790). Most participants experienced mild COVID-19 symptoms (95.74%; 95%CI: 94.17–96.91). COVID-19 severity was classified according to self-reported symptoms and medical interventions, following WHO guidelines.^18^ Mild cases included individuals with symptoms such as fever, cough, and malaise who did not require hospitalization. Moderate to severe cases were characterized by symptoms such as respiratory distress, need for supplemental oxygen, or hospitalization. However, no relationship was observed between the severity of infection and the presence of oral manifestations.

### Persistence of post-COVID-19 syndrome

The persistence of smell disorder, taste disorder, and xerostomia was analyzed for 15 days after COVID-19 recovery. The reported frequencies were 20.09% (95%CI: 17.53–22.93) for smell disorder, 12.06% (95%CI: 10.03–14.42) for taste disorder, and 5.44% (95%CI: 4.101–7.17) for xerostomia. A significant association was found between females and persistent smell disorder (p = 0.017) and taste disorder (p = 0.008) (Figure 3b).

### Oral manifestations and SARS-CoV-2 variants

**Figure 4:**
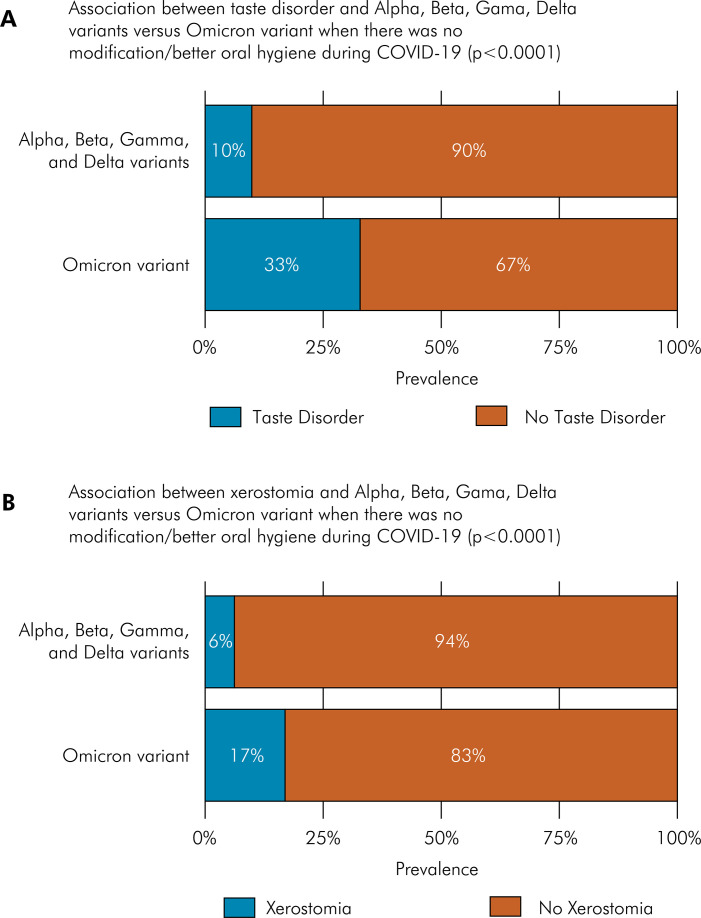
(a) Prevalence of taste disorder in comparison with the quality of oral hygiene during COVID-19 infection, considering the Alpha, Beta, Gamma, and Delta variants versus the Omicron variant. Taste disorders were less present when toothbrushing was improved or when there was no change in toothbrushing, regardless of the COVID-19 variant (p < 0.0001); (b) Prevalence of xerostomia in comparison with the quality of oral hygiene during COVID-19 infection, considering the Alpha, Beta, Gamma, and Delta variants versus the Omicron variant. Xerostomia was less present when toothbrushing was improved or when there was no change in toothbrushing, regardless of the COVID-19 variant (p < 0.0001).

When comparing the SARS-CoV-2 variants period, oral manifestations were observed in 53.62% (95%CI: 49.40–57.79) of individuals infected before December 2021 and in 58.63% (95%CI: 49.40–57.79) during the Omicron outbreak (after December 2021). The most frequent oral manifestations encountered before the emergence of the Omicron variant were taste disorder (39.89%; 95%CI: 35.84–44.08), xerostomia (16.51%; 95%CI: 16.62–19.88), and halitosis (13.17%; 95%CI: 10.58–16.29). Among participants infected during the Omicron outbreak, the most frequent oral manifestations were taste disorder (34.85%; 95%CI: 29.74–40.34), xerostomia (19.54%; 95%CI: 15.50–24.34), and difficulty in swallowing food (9.12%; 95%CI: 6.38–12.87). Considering taste disorder in participants infected during the initial outbreak, 24.12% (95%CI: 20.7–27.91%) had ageusia, 10.95% (95%CI: 8.58-13.86) presented with hypogeusia, and 4.82% (95%CI: 3.31–6.97) had dysgeusia. In contrast, among those infected during the Omicron variant outbreak, hypogeusia was observed in 14.33% (95%CI: 10.85–18.69); 13.03% (95%CI: 9.71–17.26) presented ageusia; and 7.49% (95%CI: 5.04–10.99) had dysgeusia. No significant differences were found between the samples collected before December 2021 variants and the Omicron variant when comparing oral manifestations and oral hygiene status. However, taste disorder and xerostomia were less frequent when oral hygiene was improved or remained unchanged during both periods (p <0.0001) (Figures 4a and 4b).

### Predictive variables for oral manifestations of COVID-19

The results of the logistic regression analysis indicated that several predictor variables were significantly associated with oral manifestations (pseudo R^2^ by Nagelkerke (R^2^n) = 0.159; p < 0.001), taste disorders (R^2^n = 0.147; p < 0.001), xerostomia (R^2^n = 0.120; p < 0.001), and halitosis (R^2^n = 0.204; p < 0.001). The presence of depression/anxiety significantly increased the probability of oral manifestations by about 1.86 times (OR = 1.855, 95%CI: 1.267–2.717, p = 0.002). Similarly, the presence of COVID-19 signs and symptoms was a strong predictor, increasing the probability of oral manifestations by more than 10 times (OR = 10.024, 95%CI: 1.321–76.083, p = 0.026). The presence of headaches also significantly increased the probability of oral manifestations by about 1.84 times (OR = 1.836, 95%CI: 1.349–2.497, p < 0.001). The use of medications during COVID-19 infection, especially antibiotics, increased the odds of oral manifestations by about 1.63 times (OR = 1.630, 95%CI: 1.123–2.367, p = 0.010). Poor oral hygiene quality increased the odds of oral manifestations by about 1.73 times (OR = 1.729, 95%CI: 1.189–2.516, p = 0.004). The Omicron variant increased the odds of oral manifestations by about 2.29 times (OR = 2.290, 95%CI: 1.145–4.579, p = 0.019). Lastly, being female increased the odds of oral manifestations by approximately 1.41 times compared to males, holding all other variables constant (OR = 1.4149, 95%CI: 1.00497–1.992, p = 0.047) (Table 3).

**Table 3:** Coefficients of multiple logistic regression model with all possible independent variables related to oral manifestations.

Predictor variable	Estimate	SE	Z	VIF	p-value	Odds ratio	95%CI
Intercept	-3.872	1.036	-3.74	-	< .001	0.0208	0.00273–0.159
Sex (female)	0.347	0.175	1.99	1.03	0.047	14.149	100.497–1.992
Depression/anxiety (presence)	0.618	0.195	3.17	1.03	0.002	18.550	126.667–2.717
Signs and symptoms of COVID-19 (presence)	2.305	1.034	2.23	1.01	0.026	100.235	132.054–76.083
Headache (presence)	0.607	0.157	3.87	1.03	< .001	18.357	13.4949–2.497
Antibiotics (presence)	0.488	0.155	3.14	1.01	0.002	16.296	120.196–2.209
Oral hygiene quality (worse)	0.547	0.243	2.25	1.01	0.024	17.285	107.395–2.782
SARS-CoV-2 variants (Omicron)	0.828	0.154	5.39	1.02	< .001	22.896	169.378–3.095

Regarding taste disorders, being female was marginally significant, as women were about 1.5 times more likely to develop taste disorders compared to men (OR = 1.496, 95%CI: 1.035–2.161, p = 0.032). The presence of depression/anxiety nearly doubled the likelihood of developing taste disorders (OR = 1.887, 95%CI: 1.284–2.775, p = 0.001). The presence of headaches was a strong predictor, more than doubling the likelihood of taste disorders (OR = 2.587, 95%CI: 1.858–3.601, p < 0.001). Additionally, mild disease severity increased the likelihood by about 1.5 times (OR = 1.508, 95%CI: 1.005–2.262), and the use of antibiotics increased the likelihood by about 1.4 times (OR = 1.409, 95%CI: 1.023–1.941). Regarding SARS-CoV-2 variants, infection with the Omicron variant was associated with being nearly 1.8 times more likely to develop taste disorders (OR = 1.786, 95%CI: 1.307–2.441) (Table 4).

**Table 4:** Coefficients of multiple logistic regression model with all possible independent variables related to taste disorder.

Predictor variable	Estimate	SE	Z	VIF	p-value	Odds ratio	95%CI
Intercept	-2.104	0.215	-97.863	-	< .001	0.122	0.0800–0.186
Sex (female)	0.402	0.188	21.432	1.01	0.032	1.496	10.350–2.161
Depression/Anxiety (presence)	0.635	0.197	32.293	1.01	0.001	1.887	12.835–2.775
Headache (presence)	0.950	0.169	56.302	1.01	< .001	2.587	18.581–3.601
Severity/evolution (mild)	0.411	0.207	19.841	1.01	0.047	1.508	10.050–2.262
Antibiotics (presence)	0.343	0.163	20.973	1.02	0.036	1.409	10.227–1.941
SARS-CoV-2 variants (Omicron)	0.580	0.159	36.372	1.01	< .001	1.786	13.066–2.441

Being female increased the likelihood of xerostomia by about 1.66 times compared to males (OR = 1.6643, 95%CI: 1.0361–2.6734, p = 0.035). Fever was also a significant predictor, as individuals experiencing fever were about 1.79 times more likely to develop xerostomia (OR = 1.7918, 95%CI: 1.1996–2.6763, p = 0.004). Malaise increased the likelihood of xerostomia by about 1.65 times (OR = 1.6515, 95%CI: 1.0436–2.6136, p = 0.032). The use of analgesics substantially increased the likelihood of individuals developing xerostomia by about 2.11 times (OR = 2.1096, 95%CI: 1.2947–3.4372, p = 0.003). Lastly, poor oral hygiene quality significantly elevated the likelihood of xerostomia by about 2.14 times (OR = 2.1400, 95%CI: 1.2628–3.6264, p = 0.005) (Table 5).

**Table 5:** Coefficients of multiple logistic regression model with all possible independent variables related to xerostomia.

Predictor variable	Estimate	SE	Z	VIF	p-value	Odds ratio	95%CI
Intercept	-3.207	0.313	-10.252	-	< .001	0.0405	0.0219–0.0747
Sex (female)	0.509	0.242	2.107	1.02	0.035	16.643	10.361–26.734
Fever (presence)	0.583	0.205	2.849	1.06	0.004	17.918	11.996–26.763
Malaise (presence)	0.502	0.234	2.142	1.06	0.032	16.515	10.436–26.136
Analgesic (presence)	0.746	0.249	2.997	1.05	0.003	21.096	12.947–34.372
Oral hygiene quality (worse)	0.761	0.269	2.827	1.02	0.005	21.400	12.628–36.264

Regarding halitosis, age was a significant factor, as each additional year reduced the likelihood of halitosis by about 4% (OR = 0.960, 95%CI: 0.941–0.979, p < 0.001). The presence of chronic disease and/or use of controlled medication increased the likelihood of halitosis by about 1.88 times (OR = 1.879, 95%CI: 1.170–3.016, p = 0.009). The presence of fever increased the likelihood of halitosis by about 1.75 times (OR = 1.752, 95%CI: 1.079–2.843, p = 0.023). The presence of malaise was a strong predictor, increasing the likelihood of halitosis by about 3.49 times (OR = 3.4883, 95%CI: 1.7655–6.892, p < 0.001). Mild disease severity increased the likelihood of halitosis by about 1.98 times (OR = 1.9779, 95%CI: 1.1619–3.367, p = 0.012). Worse oral hygiene also substantially increased the likelihood of halitosis by about 2.66 times (OR = 2.6632, 95%CI: 1.4649–4.842, p = 0.001). Lastly, infection with the Omicron variant significantly increased the likelihood of halitosis by about 1.88 times (OR = 1.8825, 95%CI: 1.1886–2.982, p = 0.007) (Table 6).

**Table 6:** Coefficients of multiple logistic regression model with all possible independent variables related to halitosis.

Predictor variable	Estimate	SE	Z	VIF	p-value	Odds ratio	95%CI
Intercept	-27.127	0.4746	-5.715	-	< .001	0.0664	0.0262–0.168
Age	-0.0410	0.0100	-4.094	1.09	< .001	0.9599	0.9412–0.979
Chronic disease and/or use of controlled medication (presence)	0.6305	0.2415	2.611	1.04	0.009	18.786	11.703–3.016
Fever (presence)	0.5605	0.2472	2.268	1.03	0.023	17.515	10.790–2.843
Malaise (presence)	12.494	0.3474	3.596	1.04	< .001	34.883	17.655–6.892
Severity/evolution (mild)	0.6820	0.2714	2.513	1.03	0.012	19.779	11.619–3.367
Oral hygiene quality (worse)	0.9795	0.3050	3.212	1.05	0.001	26.632	14.649–4.842
SARS-CoV-2 variants (Omicron)	0.6326	0.2346	2.696	1.01	0.007	18.825	11.886–2.982

## Discussion

Various oral manifestations associated with COVID-19 have been reported since its outbreak. In this study, the most prevalent oral manifestations were taste disorder (38%), xerostomia (17.6%), and halitosis (11.6%). Hypotheses related to the etiopathogenesis of taste disorders suggest that they may originate from an inflammatory response, a neurological response, zinc imbalance, use of medications, or a secondary response to a smell disorder.^6,19-21^ Possible mechanisms that can account for xerostomia have been indicated by some authors, including nutritional deficiency, anxiety, tension, stress, nasal congestion, salivary gland infection, hyposalivation, among others.^7,10,11,20-23^ Furthermore, it has been speculated that the use of face masks during the pandemic could promote mouth breathing, thereby inducing xerostomia and halitosis.^24^ Regarding oral lesions, authors still question whether they are caused by SARS-CoV-2 or appear as secondary manifestations.^8,25^ The results found in this study suggest a multifactorial hypothesis, given the positive association between oral manifestations, depression/anxiety, oral hygiene, medication use, and female sex.

The most widely reported comorbidities associated with COVID-19 in the literature include hypertension, diabetes, bronchial asthma, and obesity.^26^ In our study, 45.7% of the participants reported having comorbidities, among which anxiety and depression were the most frequently mentioned. Possible explanations for the increase in psychological issues include the psychiatric consequences associated with the COVID-19 situation itself. Factors such as social isolation, psychological stressors related to the immune response triggered by the virus, concerns about personal infection or transmission to family members, and the fear related to a potentially fatal new disease might have had a significant impact.^27^ Notably, there was a statistically significant association between depression and anxiety, and oral manifestations (p < 0.0001). The substantial odds ratios associated with depression and anxiety indicate a potential psychosomatic component in the manifestation of oral symptoms, suggesting that mental health interventions could be critical for affected individuals. This underscores the need to consider the profound impact of COVID-19 on mental health and to comprehend how the immune-inflammatory response translates into psychiatric diseases and oral manifestations.

In our sample, 84.16% of patients with COVID-19 required drug treatment, and medication use was associated with oral manifestations (p < 0.001). In this context, medication use may influence the oral microbiota, potentially leading to dysbiosis and adverse effects.^24^ The alteration of the oral microbiota may create an environment conducive to the development of these manifestations. Additionally, the stress and systemic effects of COVID-19, combined with the side effects of medications, could further contribute to the deterioration of oral health. Furthermore, this study showed a significant association between female sex and oral manifestations (p < 0.0001). The results of this study indicate that being female is a significant predictor for several oral manifestations in COVID-19 patients. Specifically, women were found to have a 49.6% higher probability of developing taste disorders compared to men (OR = 1.496, 95%CI: 1.035–2.161, p = 0.032). Moreover, the likelihood of xerostomia was 66.43% higher in females than in males (OR = 1.6643, 95%CI: 1.0361–2.6734, p = 0.035). These findings suggest that sex-based differences may play a crucial role in the prevalence and severity of oral symptoms associated with COVID-19. The literature suggests a possible association with the inflammatory response, as hormonal modulation and the innate immune response to viral infections appear to be more pronounced in women^28^. Also, this study observed a higher incidence of oral manifestations among individuals with deteriorating oral hygiene status (p < 0.0001). It is well established that maintaining good oral hygiene is essential for maintaining and restoring oral symbiotic balance. In addition, the COVID-19 era appears to have negatively impacted the behavioral and psychological patterns of individuals, reducing oral hygiene care.^24^ AbuBakr et al.^10^ suggested that poor oral hygiene may contribute to increased ulcerations and oral discomfort. Thus, enhancing oral hygiene practices may mitigate the risk of oral complications associated with SARS-CoV-2 infection.^29^

In the post-COVID-19 period, considering a 15-day interval after resolution of the infection, we analyzed the persistence of symptoms, including smell disorder (20.09%), taste disorder (12.06%), and xerostomia (5.44%). Notably, female individuals exhibited a higher prevalence of persistent smell disorder (p = 0.017) and persistent taste disorder (p = 0.008). The specific immune response to SARS-CoV-2 is maintained in patients with long-term COVID-19, compared to patients with complete recovery. Therefore, the persistence of viral antigens is suggested as an etiology of long COVID^30^. In our study, most participants had mild disease (95.74%) with no association between oral manifestations and the severity of the infection. Long COVID affects survivors across the full spectrum of the disease, including mild and moderate cases without the need for respiratory support or even intensive care.^31^ Reports suggest that any COVID-19 patient can develop long COVID, regardless of severity and treatment received,^32^ but some studies have identified a significant association between severity and oral manifestations.^6 33^

The present study did not show statistically significant data to support that vaccination either reduces or increases the probability of oral manifestations. The anti-SARS-CoV-2 vaccine induces a low production of SIgA by the mucous membranes, probably not being able to prevent the development of oral lesions.^34^ Furthermore, the reported prevalence of oral manifestations following vaccination was low (less than 0.31%),^35^ and orofacial adverse reactions associated with COVID-19 vaccines are still rarely reported (1:1000).^36^ Hence, vaccination stands as a secure and effective approach to disease prevention and control.^37^ A systematic review found that the Pfizer-BioNTech vaccine was linked to the highest number of oral ulcer cases (9 out of 16). However, no connection was identified between the type of vaccine or the dose administered and the occurrence of these lesions. Although the lesions were diagnosed after SARS-CoV-2 vaccination, no direct causality was established.^38^ It is important to note, however, that in Brazil, different doses of vaccines were not always consistent, with individuals often receiving different vaccine types for their first and booster doses (e.g., CoronaVac followed by Pfizer or AstraZeneca). This variability in vaccination regimens could potentially influence immune responses, including oral manifestations, but further investigation is needed to clarify these interactions. Thus, in the context of COVID-19, vaccination should be encouraged and widely distributed.

In vaccinated populations, Omicron appears to cause less severe acute illness than previous variants.^14^ Some studies report oral manifestations in participants infected with the Omicron variant; however, there are few distinctions between the other variants. Before December 2021, smell and taste disorders were more frequent, whereas the prevalence of loss of smell decreased in cases of the Omicron variant.^39,40^ Concerning taste disorder and xerostomia, for variants before December 2021, there was a statistically significant difference between patients with COVID-19 and those who recovered.^5,13^ But, with Omicron, fever, dry cough, and sore throat are more frequently reported.^41^ In our study, oral manifestations were quite similar to those observed in previous variants (53.62%) and during the Omicron outbreak (58.63%). A slight variation was also seen for taste disorders (from 34.85% to 38.89%) and xerostomia (from 16.51% to 19.54%). The literature reports a reduced prevalence of chemosensory dysfunction by Omicron, which underscores the need to investigate the differences in prevalence and severity of chemosensory dysfunction, depending on the variant.^39^

This study has several limitations. First, while we achieved the optimal sample size, it was obtained for convenience through digital media and networks. Second, despite the pre-assessment with internal validity, the reliability of the questionnaire was not measured by Cronbach’s alpha, and an external validity cannot be determined. Third, questionnaire-based studies depend on the participants’ subjective interpretation , which can compromise the findings. Fourth, as this is a cross-sectional study, there is an inherent limitation of reverse causality. Fifth, this is a self-perception study, so there was no clinical examination of patients. Sixth, genomic sequencing to identify the virus variant was not conducted in this study. While there is a higher chance of exposure to a specific variant during its peak prevalence, it is acknowledged that this method is not the most accurate for determining the specific variant responsible for an individual’s infection. Seventh, the small number of non-vaccinated individuals (2.12% of the sample) limited our ability to draw robust comparisons between vaccinated and non-vaccinated populations. No significant differences were observed between these groups, and future research with larger and more balanced sample sizes is necessary to better understand any potential differences in oral manifestations. Finally, there is no consensus in the literature on which interval should be considered to characterize the “post-COVID-19 syndrome”, which can range from 15 days to more than 12 weeks.^17^ Despite these limitations, this research is innovative and unprecedented in exploring the different oral scenarios of COVID-19, including variants, time periods and vaccination. The questionnaires were especially developed and adapted for the Brazilian population, considering the lack of standard questionnaires for COVID-19. In addition, a sufficient sample size and power were obtained.

## Conclusion

In this study, we observed a significant prevalence of oral manifestations among participants with COVID-19, particularly taste disorders, xerostomia, and halitosis. Our findings indicate that these manifestations were more prevalent among individuals experiencing symptoms of depression and anxiety, as well as among those with poorer oral hygiene. Notably, the emergence of the Omicron variant was associated with an increased likelihood of oral complications. Additionally, the use of medications, particularly antibiotics, may further impact oral health, leading to additional complications. While most participants reported mild symptoms of COVID-19, the persistence of oral symptoms such as taste and smell disorders post-recovery highlights the need for ongoing monitoring and care for affected individuals. Overall, this study underscores the importance of assessing oral health in COVID-19 and suggests that mental health, hygiene practices, and medication use may be critical in mitigating oral complications during and after infection.

## Data Availability

The authors declare that all data generated or analyzed during this study are included in this published article.
